# NeoFox: annotating neoantigen candidates with neoantigen features

**DOI:** 10.1093/bioinformatics/btab344

**Published:** 2021-05-10

**Authors:** Franziska Lang, Pablo Riesgo-Ferreiro, Martin Löwer, Ugur Sahin, Barbara Schrörs

**Affiliations:** Biomarker Development Center, TRON–Translational Oncology at the University Medical Center of the Johannes Gutenberg University Mainz, 55131 Mainz, Germany; Biomarker Development Center, TRON–Translational Oncology at the University Medical Center of the Johannes Gutenberg University Mainz, 55131 Mainz, Germany; Biomarker Development Center, TRON–Translational Oncology at the University Medical Center of the Johannes Gutenberg University Mainz, 55131 Mainz, Germany; Research Center for Immunotherapy (FZI), University Medical Center of the Johannes Gutenberg University Mainz, 55131 Mainz, Germany; CEO, BioNTech SE, Mainz, Germany; Biomarker Development Center, TRON–Translational Oncology at the University Medical Center of the Johannes Gutenberg University Mainz, 55131 Mainz, Germany

## Abstract

**Summary:**

The detection and prediction of true neoantigens is of great importance for the field of cancer immunotherapy. Wesearched the literature for proposed neoantigen features and integrated them into a toolbox called NEOantigen Feature toolbOX (NeoFox). NeoFox is an easy-to-use Python package that enables the annotation of neoantigen candidates with 16 neoantigen features.

**Availability and implementation:**

NeoFox is freely available as an open source Python package released under the GNU General Public License (GPL) v3 license at https://github.com/TRON-Bioinformatics/neofox.

**Supplementary information:**

Supplementary data are available at *Bioinformatics* online.

## 1 Introduction

Somatic mutations can generate mutated gene products, so called neoantigens that are able to drive anti-tumoral immune responses. Their break down products (neoepitopes) are presented on MHC (major histocompatibility complex) molecules and are recognized by CD4^+^ or CD8^+^ T cells. The success of many cancer immunotherapies depends on the anti-tumoral effect of such neoantigen-specific immune responses (Sahin and Türeci, 2018). Importantly, the success of neoantigen vaccination relies on the selection of true neoantigens from the individual neoantigen profile of cancer patients. Several algorithms and neoantigen features that might underlie immunogenicity have been published and are already in use for target prioritization. Recent efforts undertaken by the TESLA (Tumor nEoantigen SeLection Alliance) consortium highlight the importance of considering multiple biological aspects of neoantigens (Wells *et al.*, 2020). Here, we introduce NeoFox as a NEOantigen Feature ToolbOX to annotate neoantigen candidates with 16 neoantigen features. NeoFox bridges biology and bioinformatics by creating a biological meaningful representation of the neoantigen recognition process.

## 2 NeoFox software tool

### 2.1 Neoantigen features

We searched the literature for algorithms that are used to prioritize neoantigen candidates for their potential to elicit T-cell responses. Here, we focus on algorithms that represent single features of neoantigens or combinations of single features. Included algorithms cover several aspects of a neoantigen and were integrated into NeoFox (see Supplementary Information for tool design) or alternatively were re-implemented based on descriptions in the original publication (Supplementary Table S1).

Being expressed and presented on the cell surface of tumor or antigen-presenting cells is the pre-requisite for a mutation to be recognized by T cells. RNA expression data may not be available in all cases. However, expression is an important feature of neoantigens and was already used successfully for target prioritization (Sahin *et al.,* 2017). Therefore, we support the annotation of neoantigen candidates with median gene expression in the TCGA subcohort of the respective cancer entity if no patient-specific transcript expression is available.

**Fig. 1.**
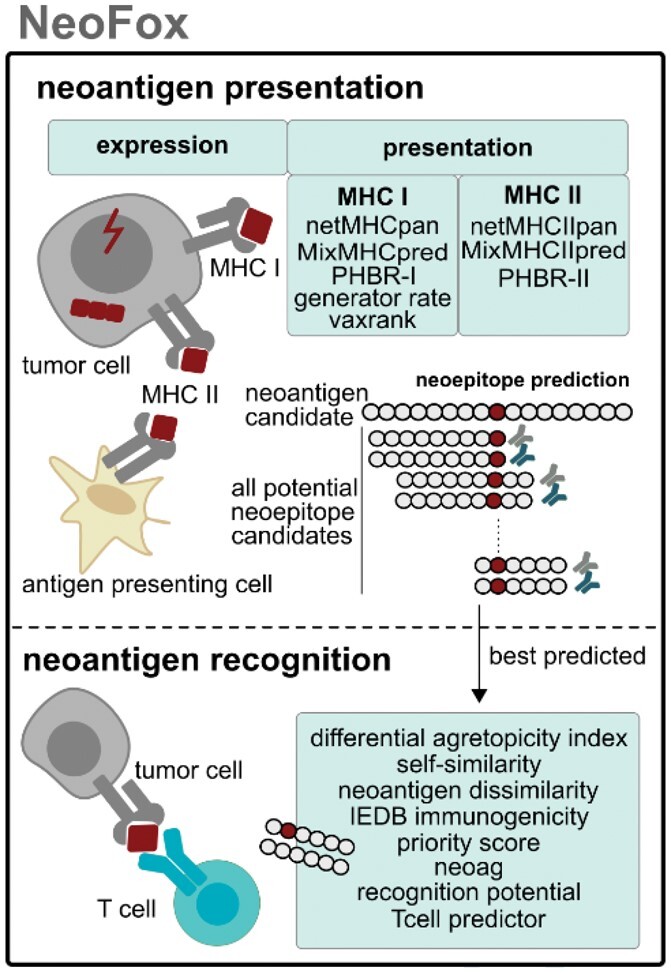
Neoantigen features implementend in NeoFox tool. NeoFox annotates neoantigen candidates with features that are related to presentation or recognition. To model neoantigen presentation, neoepitope candidates are predicted covering all potential epitope lengths and HLA alleles. The best predicted MHC I neoepitope candidate serves as a basis to calculate neoantigen features that model neoantigen recognition

The ability of a neoantigen candidate to be presented to T cells is estimated by multiple approaches, while considering all MHC alleles of the patient and epitope lengths that are supported by the respective approach(Fig. 1, Supplementary Information). MHC I neoepitope candidates are predicted with the MHC I binding predictor netMHCpan (Jurtz et al., 2017) using both IC50 and percentile rank and with the MHC I ligand predictor MixMHCpred (Bassani-Sternberg et al., 2017) using rank and score. Likewise, MHC II neoepitope candidates are predicted with netMHCIIpan (Jensen et al., 2018) and MixMHC2pred (Racle et al., 2019). For each of these methods, NeoFox returns the best predicted neoepitope candidate with predicted binding value and the corresponding MHC allele per provided neoantigen candidate. This best predicted approach is complemented by estimations on whether a neoantigen candidate can be presented multiple times which potentially increases the likelihood of T-cell recognition. Additional features go beyond presentation modeling and estimate the likelihood of T-cell recognition (Fig. 1). These features model the foreignness of the best predicted neoepitope per neoantigen candidate by comparing the amino acid sequence to wild-type (WT) or pathogen sequences. Other approaches combine aspects such as sequence characteristics by *ad hoc* or machine learning models.

### 2.2 Usage

NeoFox can be used as a command line tool or programmatically and requires two types of inputs: neoantigen candidates and patient data. The first one requires the neoantigen candidate sequence, its corresponding WT sequence and gene name (Supplementary Table S2). Furthermore, RNA expression and RNA and DNA variant allele frequencies are optional. Expression values are not expected in a specific format but they should be comparable across candidates. Currently, only neoantigen candidates derived from point mutations are supported. The patient data contains the MHC alleles of the patients and optionally the tumor type (Supplementary Table S3). Neoantigen candidates are returned with annotated features, while appending user-specific information in the neoantigen candidate input as additional annotations (Supplementary Tables S2 and S4).

## 3 Discussion

Several pipelines that predict neoantigen candidates from mutation lists are published (Hundal et al., 2020; Kodysh and Rubinsteyn, 2020). However, these pipelines usually focus on a selection of features to rank neoantigen candidates. A tool that provides a comprehensive description of neoantigen candidates by proposed features is still missing and NeoFox closes this gap. Importantly, we intend to cover future developments in the field of neoantigen prioritization by continuous extension of the tool with new features. Besides comprehensive feature annotation, NeoFox has several other advantageous properties: (i) it can be run from the command line or easily embedded into existing pipelines using the Python API (application programming interface). (ii) Several input and output formats are supported and can be selected by the user. (iii) The data models are close to biology and intuitive, (iv) but are flexible and support user-specific annotations in the input data.

Given the small number of large-scale immunogenicity datasets, NeoFox will be a valuable resource for annotating future datasets with neoantigen features to investigate their ability to describe T-cell responses. NeoFox could be integrated into automated processing, saving predicted neoantigen candidates into databases upon storage for large-scale analysis.

## Supplementary Material

btab344_Supplementary_Data
