# Correction to: NeoFox: annotating neoantigen candidates with neoantigen features

**DOI:** 10.1093/bioinformatics/btad763

**Published:** 2023-12-22

This is a correction to: Franziska Lang, Pablo Riesgo-Ferreiro, Martin Löwer, Ugur Sahin, Barbara Schrörs, NeoFox: annotating neoantigen candidates with neoantigen features, *Bioinformatics*, Volume 37, Issue 22, November 2021, Pages 4246–4247, https://doi.org/10.1093/bioinformatics/btab344

In the originally published version of this manuscript, author Franziska Lang’s second affiliation was omitted in error.

Franziska Lang’s affiliations should have been given as:

“Biomarker Development Center, TRON–Translational Oncology at the University Medical Center of the Johannes Gutenberg University Mainz, 55131 Mainz, Germany” and “Faculty of Biology, Johannes Gutenberg University Mainz, 55128 Mainz, Germany”.

These details have been corrected only in this correction notice to preserve the published version of record.

