# Supplementary material for: NeoFox: annotating neoantigen candidates with neoantigen features
Source: Bioinformatics. 2021 May 10;37(22):4246–7. doi: 10.1093/bioinformatics/btab344 (PMC9502226; doi:10.1093/bioinformatics/btab344)
Supplement: btab344_Supplementary_Data [file btab344_supplementary_data.docx]

Supplementary Information:

**NeoFox: annotating neoantigen candidates with neoantigen features**

Franziska Lang^1†^, Pablo Riesgo Ferreiro^1†^, Martin Löwer^1^, Ugur Sahin^2,3^, Barbara Schrörs^1*^

^1^TRON–Translational Oncology at the University Medical Center of the Johannes Gutenberg University Mainz, Germany; ^2^ University Medical Center of the Johannes Gutenberg University Mainz, Germany, ^3^BioNTech SE, Mainz, Germany, † equal contribution

^†^equal contribution

* To whom correspondence should be addressed: Barbara.Schroers@TrOn-Mainz.DE

**Supplementary Text**

## Design

NeoFox is implemented in the Python programming language. Biological entities such as neoantigens and patients are modelled using Protocol buffers (Supplementary Figure S1). These models are characterized by minimal requirements but are extensible if a user has more information such as experimental immunogenicity information. These models foster data normalization and validity, while minimizing data heterogeneity. The data models serve as a lingua franca to represent the neoantigen recognition process enabling machine readability. NeoFox annotates neoantigen candidates by accessing neoantigen features that are organized in a modular architecture. Importantly, some features rely on results from other features. For example, the best neoepitope candidate based on MHC I binding prediction with netMHCpan per neoantigen candidate is used for other features. This means that MHC I binding scores are determined for all possible 8-11mers that cover the mutation against all provided MHC I alleles. The mutated epitope with the lowest MHC binding rank over all kmer/MHC I allele combinations is selected as the best candidate.

To allow processing in a timely manner, parallel annotation of neoantigen candidates is implemented (see Benchmarking).

## Benchmarking

We benchmarked the performance of NeoFox with a synthetic dataset. The synthetic dataset of neoantigen candidates was built by randomly taking 27-mers from the proteome. The middle amino acid (position 14) was mutated to generate pairs of neoantigen candidates and corresponding wild type (WT) sequences. Random expression values were generated for each neoantigen candidate. The synthetic dataset of patients was generated by sampling MHC alleles from known MHC alleles to build synthetic MHC I and MHC II genotypes.

We benchmarked the performance of neoantigen candidate datasets with an input size of 10, 100, 1000 and 10,000 data points, using 1, 5, 10 or 50 CPUs (central processing unit) (Supplemental Figure S2, Supplemental Table 5). A single neoantigen candidate can be processed with a single CPU in 20 seconds (+/- 6 sec). No significant overhead due to parallelization was observed. The annotation of 100 neoantigen candidates with 10 CPUs takes ~2.88 minutes while using ~303 MB. The memory use increases to up to 2.5 GB when processing a dataset of 10,000 neoantigens candidates.

**Supplementary Table 1**: Description of neoantigen features implemented in NeoFox. MHC: Major histocompatibility complex, DAI: Differential Agretopicity Index, WT: wild type, BLOSUM: Blocks Substitution Matrix, IEDB: Immune Epitope Database, BLAST: Basic Local Alignment Search Tool, PHBR: Patient Harmonic-mean Best Rank, VAF: variant allele frequency, * by netMHCpan

| **Feature** | **Method** | **Description** | **Installation required?** | **Dependent on netMHCpan** | **Input** | **Reference** | **Comment** |
| --- | --- | --- | --- | --- | --- | --- | --- |
| **MHC I binding/presentation** | **MHC I binding affinity/rank score (netMHCpan-v4.0)** | MHC I binding prediction using a neural network trained on binding affinity and eluted ligands | yes | - | Neoantigen candidate sequence, MHC I alleles | Jurtz *et al.*, 2017 | Best predicted per neoantigen candidate is returned, Neural network available as web server |
|  | **MixMHCpred score v2.1** | Prediction of cell surface presentation by MHC I using a motif deconvolution algorithm trained on eluted ligands for | yes | no | Neoepitope candidate sequence, MHC I alleles | Bassani-Sternberg *et al.*, 2017; Gfeller *et al.*, 2018 | Best predicted per neoantigen candidate is returned, Trained on eluted ligands detected with mass spectrometry |
|  | **PHBR-I** | Ability to present a particular mutation considering all MHC I alleles of a patient | no | yes | Best MHC I binding rank score for each patient MHC I allele | Marty *et al.*, 2017 | Harmonic mean of MHC I rank scores |
|  | **Generator rate** | Ability to present a particular mutation by multiple neoepitopes | no | yes | MHC I binding affinities | Rech *et al.*, 2018 | In the original paper, two thresholds to define neoepitopes were presented. Here, the generator rate is calculated based on the threshold for conserved defined neoepitopes: MHC I binding affinity < 50 nM. |
| **MHC II binding/presentation** | **MHC II binding affinity/rank score (netMHCIIpan-v3.2)** | MHC II binding prediction using a neural network trained on binding affinity and eluted ligands | yes | no | Neoantigen candidate sequence, MHC II alleles | Jensen *et al.*, 2018 | Best predicted per neoantigen candidate is returned , Neural network available as web server |
|  | **MixMHC2pred score v1.2** | Prediction of cell surface presentation by MHC II using a motif deconvolution algorithm trained on eluted ligands for | yes | no | Neoepitope candidate sequence, MHC II alleles | Racle *et al.*, 2019 | Best predicted per neoantigen candidate is returned, Trained on eluted ligands detected with mass spectrometry |
|  | **PHBR-II** | Ability to present a particular mutation considering all MHC II alleles of a patient | no | yes | Best MHC II binding rank score for each patient MHC II allele | Marty Pyke *et al.*, 2018 | Harmonic mean of MHC I rank scores |
| **Similarity between mutated and self** | **Differential Agretopicity Index (DAI)** | Difference in MHC I binding affinities between mutated epitope and WT peptide | no | yes | MHC I binding affinity of best predicted neoepitope candidate and corresponding WT epitope | Duan *et al.*, 2014; Ghorani *et al.*, 2017. | - |
|  | **Self-Similarity** | Sequence similarity between mutated and WT peptide using a kernel similarity measure (Shen *et al.*, 2012). | no | yes | Best predicted neoepitope candidate sequence, corresponding WT peptide sequence | Bjerregaard *et al.*, 2017a | In short, all kmers of the mutated epitope are compared to all kmers in the WT peptide using a BLOSUM similarity measure. Was found to be specifically relevant for neoepitopes with similar MHC binding as WT epitope |
|  | **Neoantigen dissimilarity** | Dissimilarity of neoepitope candidate to self-proteome | no | yes | Best predicted neoepitope candidate sequence, WT proteome | Richman *et al.*, 2019 | BLAST search against WT proteome |
| **Sequence preferences** | **IEDB immunogenicity** | Summed up position-weighted enrichment of residues of the neoepitope candidate in immunogenic peptide sequences | no | yes | Best predicted neoepitope candidate sequence | Calis *et al.*, 2013 | - |
| **Combination of features** | **Recognition potential** | Combination of similarity to known pathogen epitopes and differential MHC binding of neoepitope candidate and WT peptide | no | yes | Best predicted neoepitope candidate sequence, MHC I binding affinities of best predicted neoepitope candidate and corresponding WT peptide | Łuksza *et al.*, 2017; Balachandran *et al.*, 2017 | Best 9mer epitope is used here, BLAST search against IEDB database |
|  | **Vaxrank** | Combination of the ability of a mutation to be presented by multiple epitopes and mutated transcript expression | no | yes | Transcript expression of mutation, MHC I binding affinities of neoepitope candidates related to the neoantigen candidate | Rubinsteyn *et al.*, 2018; Kodysh and Rubinsteyn, 2020 | In the original publication, the number of reads supporting the mutations is used. NeoFox uses the product of transcript expression and VAF. |
|  | **Priority score** | Combination of MHC binding of the neoepitope candidate and corresponding WT peptide, expression and VAF | no | yes | MHC I binding score of best predicted neoepitope candidate, MHC I binding score of corresponding WT peptide, transcript expression, variant allele frequency | Bjerregaard *et al.*, 2017b. | - |
|  | **Tcell predictor** | Random Forest Model considering several neoepitope candidate features such as expression, hydrophobicity | no | yes | Gene name, substitution, best predicted neoepitope candidate sequence | Besser *et al.*, 2019 | Best 9mer epitope is used here |
|  | **neoag** | Gradient Boosting Model considering multiple features that are intrinsic to the neoepitope candidate | no | yes | Best predicted neoepitope candidate sequence, corresponding WT peptide, variant position, MHC I binding | Smith *et al.*, 2019 | Trained on a immunogenicity dataset from mouse tumor models |

**Supplementary Table 2**: Example of an input table with neoantigen candidates. The columns gene, mutation.mutatedXmer (mutated peptide sequence), mutation.wildTypeXmer (corresponding WT sequence) and patientIdentifier are mandatory. rnaExpression, rnaVariantAlleleFrequency, dnaVariantAlleleFrequency are optional. External user-specific annotations can be added and will be returned in the output table. rnaExpression can be provided in user-preferred format (e.g. TPM or RPKM) but should be consistent. If rnaExpression is not provided for all neoantigen candidates of a patient, these are annotated with gene expression in the respective subcohort of TCGA. More details are provided in the online documentations of NeoFox: https://neofox.readthedocs.io

| **gene** | **mutation.wildTypeXmer** | **mutation.mutatedXmer** | **patientIdentifier** | **rnaExpression** | **rnaVariantAlleleFrequency`** | **dnaVariantAlleleFrequency** | **external_annotation_1** | **external_annotation_2** |
| --- | --- | --- | --- | --- | --- | --- | --- | --- |
| BRCA2 | AAAAAAAAAAAAALAAAAAAAAAAAAA | AAAAAAAAAAAAAFAAAAAAAAAAAAA | Ptx | 7.942 | 0.85 | 0.34 | some_value | some_value |
| BRCA2 | AAAAAAAAAAAAAMAAAAAAAAAAAAA | AAAAAAAAAAAAARAAAAAAAAAAAAA | Ptx | 7.942 | 0.85 | 0.34 | some_value | some_value |
| BRCA2 | AAAAAAAAAAAAAGAAAAAAAAAAAAA | AAAAAAAAAAAAAKAAAAAAAAAAAAA | Ptx | 7.942 | 0.85 | 0.34 | some_value | some_value |

**Supplementary Table 3**: Example of an input table with patient information. This table requires the following columns: identifier (of the patient), mhcIAlleles (comma-separated MHC I alleles of the patient, mhcIIAlleles (comma-separated mhcIIAlleles). tumorType (tumor entity in the TCGA study abbreviation format) is optional.

| **identifier** | **mhcIAlleles** | **mhcIIAlleles** | **tumorType** |
| --- | --- | --- | --- |
| Ptx | HLA-A*03:01,HLA-A*29:02,HLA-B*07:02,HLA-B*44:03,HLA-C*07:02,HLA-C*16:01 | HLA-DRB1*04:02,HLA-DRB1*08:01,HLA-DQA1*03:01,HLA-DQA1*04:01,HLA-DQB1*03:02,HLA-DQB1*04:02,HLA-DPA1*01:03,HLA-DPA1*02:01,HLA-DPB1*13:01,HLA-DPB1*04:01 | HNSC |
| Pty | HLA-A*03:01,HLA-A*29:02,HLA-B*07:02,HLA-B*44:03,HLA-C*07:02,HLA-C*16:01 | HLA-DRB1*04:02,HLA-DRB1*08:01,HLA-DQA1*03:01,HLA-DQA1*04:01,HLA-DQB1*03:02,HLA-DQB1*04:02,HLA-DPA1*01:03,HLA-DPA1*02:01,HLA-DPB1*13:01,HLA-DPB1*04:01 | HNSC |

**Supplementary Table 4**: Example of an output file with annotated neoantigen candidates based on Supplementary Table 2 and Supplementary Table 3 as input. Each row in the tabular format represents one neoantigen candidate. Each neoantigen candidate will receive a unique identifier. More details are provided in the online documentations of NeoFox: https://neofox.readthedocs.io

| **identifier** | **dnaVariantAlleleFrequency** | **gene** | **mutation.mutatedXmer** | **mutation.position** | **mutation.wildTypeXmer** | **patientIdentifier** |
| --- | --- | --- | --- | --- | --- | --- |
| ou11p7RD+tZvjY88DA55Mw== | 0.294 | BRCA2 | AAAAAAAAAAAAAFAAAAAAAAAAAAA | 14 | AAAAAAAAAAAAALAAAAAAAAAAAAA | Ptx |
| rzXB3nQlZ5misn6VN8EA2A== | 0.173 | BRCA2 | AAAAAAAAAAAAAMAAAAAAAAAAAAA | 14 | AAAAAAAAAAAAARAAAAAAAAAAAAA | Ptx |

| **rnaExpression** | **rnaVariantAlleleFrequency** | **imputedGeneExpression** | **external_annotation_1** | **external_annotation_2** | **ADN_MHCI** | **ADN_MHCII** | **Amplitude_MHCII_rank** | **Amplitude_MHCI_affinity** |
| --- | --- | --- | --- | --- | --- | --- | --- | --- |
| 0.51950689 | 0.857 | 2.2 | some_value | some_value | 0 | 1 | 28 | 0.88723 |
| 0.51950689 | 0.556 | 2.2 | some_value | some_value | 1 | 1 | 10 | 90.685 |

| **Amplitude_MHCI_affinity_9mer** | **Best_affinity_MHCII_allele** | **Best_affinity_MHCII_allele_WT** | **Best_affinity_MHCII_epitope** | **Best_affinity_MHCII_epitope_WT** | **Best_affinity_MHCII_score** | **Best_affinity_MHCII_score_WT** |
| --- | --- | --- | --- | --- | --- | --- |
| 0.88723 | HLA-DQA10401-DQB10402 | HLA-DQA10401-DQB10402 | AAAAFAAAAAAAAAA | AAAALAAAAAAAAAA | 251.77 | 513.02 |
| 90.685 | HLA-DQA10401-DQB10402 | HLA-DQA10401-DQB10402 | AAAAAAAAAMAAAAA | AAAAAAAAARAAAAA | 421.53 | 554.92 |

| **Best_affinity_MHCI_9mer_allele** | **Best_affinity_MHCI_9mer_allele_WT** | **Best_affinity_MHCI_9mer_anchor_mutated** | **Best_affinity_MHCI_9mer_epitope** | **Best_affinity_MHCI_9mer_epitope_WT** | **Best_affinity_MHCI_9mer_position_mutation** | **Best_affinity_MHCI_9mer_score** |
| --- | --- | --- | --- | --- | --- | --- |
| HLA-C*16:01 | HLA-C*16:01 | 1 | AAAAAAAAF | AAAAAAAAL | 9 | 24.3 |
| HLA-C*16:01 | HLA-C*16:01 | 1 | AAAAAAAAM | AAAAAAAAR | 9 | 24.1 |

| **Best_affinity_MHCI_9mer_score_WT** | **Best_affinity_MHCI_allele** | **Best_affinity_MHCI_allele_WT** | **Best_affinity_MHCI_epitope** | **Best_affinity_MHCI_epitope_WT** | **Best_affinity_MHCI_score** | **Best_affinity_MHCI_score_WT** |
| --- | --- | --- | --- | --- | --- | --- |
| 21.7 | HLA-C*16:01 | HLA-C*16:01 | AAAAAAAAF | AAAAAAAAL | 24.3 | 21.7 |
| 6346.9 | HLA-C*16:01 | HLA-C*16:01 | AAAAAAAAM | AAAAAAAAR | 24.1 | 6346.9 |

| **Best_rank_MHCII_score** | **Best_rank_MHCII_score_WT** | **Best_rank_MHCII_score_allele** | **Best_rank_MHCII_score_allele_WT** | **Best_rank_MHCII_score_epitope** | **Best_rank_MHCII_score_epitope_WT** | **Best_rank_MHCI_9mer_allele** |
| --- | --- | --- | --- | --- | --- | --- |
| 0.05 | 1.4 | HLA-DQA10301-DQB10402 | HLA-DQA10301-DQB10402 | AAAAFAAAAAAAAAA | AAAALAAAAAAAAAA | HLA-C*16:01 |
| 0.25 | 2.5 | HLA-DQA10401-DQB10302 | HLA-DQA10401-DQB10302 | AAAAAAAAAAMAAAA | AAAAAAAAAARAAAA | HLA-C*16:01 |

| **Best_rank_MHCI_9mer_allele_WT** | **Best_rank_MHCI_9mer_epitope** | **Best_rank_MHCI_9mer_epitope_WT** | **Best_rank_MHCI_9mer_score** | **Best_rank_MHCI_9mer_score_WT** | **Best_rank_MHCI_score** | **Best_rank_MHCI_score_WT** |
| --- | --- | --- | --- | --- | --- | --- |
| HLA-C*16:01 | AAAAAAAAF | AAAAAAAAL | 0.0592 | 0.0493 | 0.0592 | 0.0493 |
| HLA-C*16:01 | AAAAAAAAM | AAAAAAAAR | 0.0587 | 8.9317 | 0.0587 | 8.9317 |

| **Best_rank_MHCI_score_allele** | **Best_rank_MHCI_score_allele_WT** | **Best_rank_MHCI_score_epitope** | **Best_rank_MHCI_score_epitope_WT** | **CDN_MHCI** | **CDN_MHCII** | **DAI_MHCI_affinity_cutoff500nM** |
| --- | --- | --- | --- | --- | --- | --- |
| HLA-C*16:01 | HLA-C*16:01 | AAAAAAAAF | AAAAAAAAL | 1 | 1 | -2.6 |
| HLA-C*16:01 | HLA-C*16:01 | AAAAAAAAM | AAAAAAAAR | 1 | 1 | 6322.8 |

| **Dissimilarity_MHCI_cutoff500nM** | **Expression_mutated_transcript** | **Generator_rate** | **IEDB_Immunogenicity_MHCI_cutoff500nM** | **Improved_Binder_MHCI** | **MixMHC2pred_best_allele** | **MixMHC2pred_best_peptide** |
| --- | --- | --- | --- | --- | --- | --- |
| 1 | 0.44522 | 1 | 0.18288 | 0 | DPA1_01_03__DPB1_04_01 | AAAAFAAAAAAAAAAA |
| 1 | 0.39796 | 1 | 0.18288 | 1 | DPA1_01_03__DPB1_04_01 | AAAAMAAAAAAAAAAA |

| **MixMHC2pred_best_rank** | **MixMHCpred_best_allele** | **MixMHCpred_best_peptide** | **MixMHCpred_best_rank** | **MixMHCpred_best_score** | **Neoag_immunogenicity** | **Number_of_mismatches_MCHI** |
| --- | --- | --- | --- | --- | --- | --- |
| 0.997 | B0702 | AAAAAAAAF | 0.1 | 0.50487 | 13.16998 | 1 |
| 2.44 | B0702 | AAAAAAAAM | 0.07 | 0.5444 | 39.51379 | 1 |

| **PHBR-I** | **PHBR-II** | **Pathogensimiliarity_MHCI_affinity_9mer** | **Priority_score** | **Recognition_Potential_MHCI_affinity_9mer** | **Selfsimilarity_MHCI_conserved_binder** | **Tcell_predictor_score_cutoff500nM** |
| --- | --- | --- | --- | --- | --- | --- |
| 0.31193 | 0.21892 | 0 | 0.07017 | 0 | 0.99178271 | 0.40327581 |
| 0.29303 | 1.5594 | 0 | 0.10626 | 0 | NA | 0.46452844 |

| **VAF_in_RNA** | **VAF_in_tumor** | **mutation_not_found_in_proteome** | **patient** | **substitution** | **transcript_expression** | **vaxrank_binding_score** | **vaxrank_total_score** |
| --- | --- | --- | --- | --- | --- | --- | --- |
| 0.857 | 0.294 | 1 | Ptx | I547T | 0.51950689 | 3.7689 | 1.678 |
| 0.556 | 0.173 | 1 | Ptx | E135S | 0.71575659 | 3.8741 | 1.5417 |

**Supplementary Table 5**: Performance evaluation of NeoFox 10, 100, 1000 and 10000 neoantigen candidates with 1- 50 CPUs. Each measurement was repeated at least 3x. CPU: central processing unit.

| # neoantigen candidates | # cpus | replicate | run time | memory in kilobytes |
| --- | --- | --- | --- | --- |
| 10 | 1 | 0 | 2:28.79 | 269796 |
| 10 | 1 | 1 | 2:20.90 | 270060 |
| 10 | 1 | 2 | 2:30.56 | 268676 |
| 10 | 1 | 3 | 2:17.82 | 270640 |
| 10 | 1 | 4 | 2:17.24 | 269260 |
| 10 | 5 | 0 | 0:40.73 | 296852 |
| 10 | 5 | 1 | 0:38.24 | 295996 |
| 10 | 5 | 2 | 0:40.20 | 299876 |
| 10 | 5 | 3 | 0:37.61 | 297632 |
| 10 | 5 | 4 | 0:37.39 | 295632 |
| 10 | 10 | 0 | 0:27.47 | 293972 |
| 10 | 10 | 1 | 0:25.95 | 296376 |
| 10 | 10 | 2 | 0:26.49 | 296092 |
| 10 | 10 | 3 | 0:26.44 | 292104 |
| 10 | 10 | 4 | 0:25.32 | 304552 |
| 100 | 1 | 0 | 23:37.87 | 335280 |
| 100 | 1 | 1 | 23:43.26 | 333824 |
| 100 | 1 | 2 | 23:22.82 | 333760 |
| 100 | 1 | 3 | 23:49.56 | 333896 |
| 100 | 1 | 4 | 23:40.46 | 334612 |
| 100 | 5 | 0 | 5:16.18 | 311800 |
| 100 | 5 | 1 | 5:15.54 | 315316 |
| 100 | 5 | 2 | 5:14.90 | 314436 |
| 100 | 5 | 3 | 5:18.18 | 310052 |
| 100 | 5 | 4 | 5:14.50 | 311396 |
| 100 | 10 | 0 | 2:53.99 | 303736 |
| 100 | 10 | 1 | 2:53.43 | 306328 |
| 100 | 10 | 2 | 2:53.74 | 301640 |
| 100 | 10 | 3 | 2:55.72 | 302916 |
| 100 | 10 | 4 | 2:53.22 | 305836 |
| 100 | 50 | 0 | 1:09.13 | 303444 |
| 100 | 50 | 1 | 1:09.67 | 304144 |
| 100 | 50 | 2 | 1:08.09 | 303228 |
| 100 | 50 | 3 | 1:10.56 | 301272 |
| 100 | 50 | 4 | 1:08.42 | 302336 |
| 1000 | 10 | 0 | 35:35.77 | 381968 |
| 1000 | 10 | 1 | 26:42.36 | 371160 |
| 1000 | 10 | 2 | 26:43.46 | 369840 |
| 1000 | 10 | 3 | 26:29.09 | 369572 |
| 1000 | 10 | 4 | 26:40.56 | 370136 |
| 1000 | 50 | 0 | 7:29.89 | 350528 |
| 1000 | 50 | 1 | 7:23.62 | 353296 |
| 1000 | 50 | 2 | 7:24.01 | 350988 |
| 1000 | 50 | 3 | 7:16.30 | 343748 |
| 1000 | 50 | 4 | 7:11.44 | 347340 |
| 10000 | 50 | 0 | 01:07:32 | 2311952 |
| 10000 | 50 | 1 | 01:08:12 | 2300208 |
| 10000 | 50 | 2 | 01:07:40 | 2293104 |

**
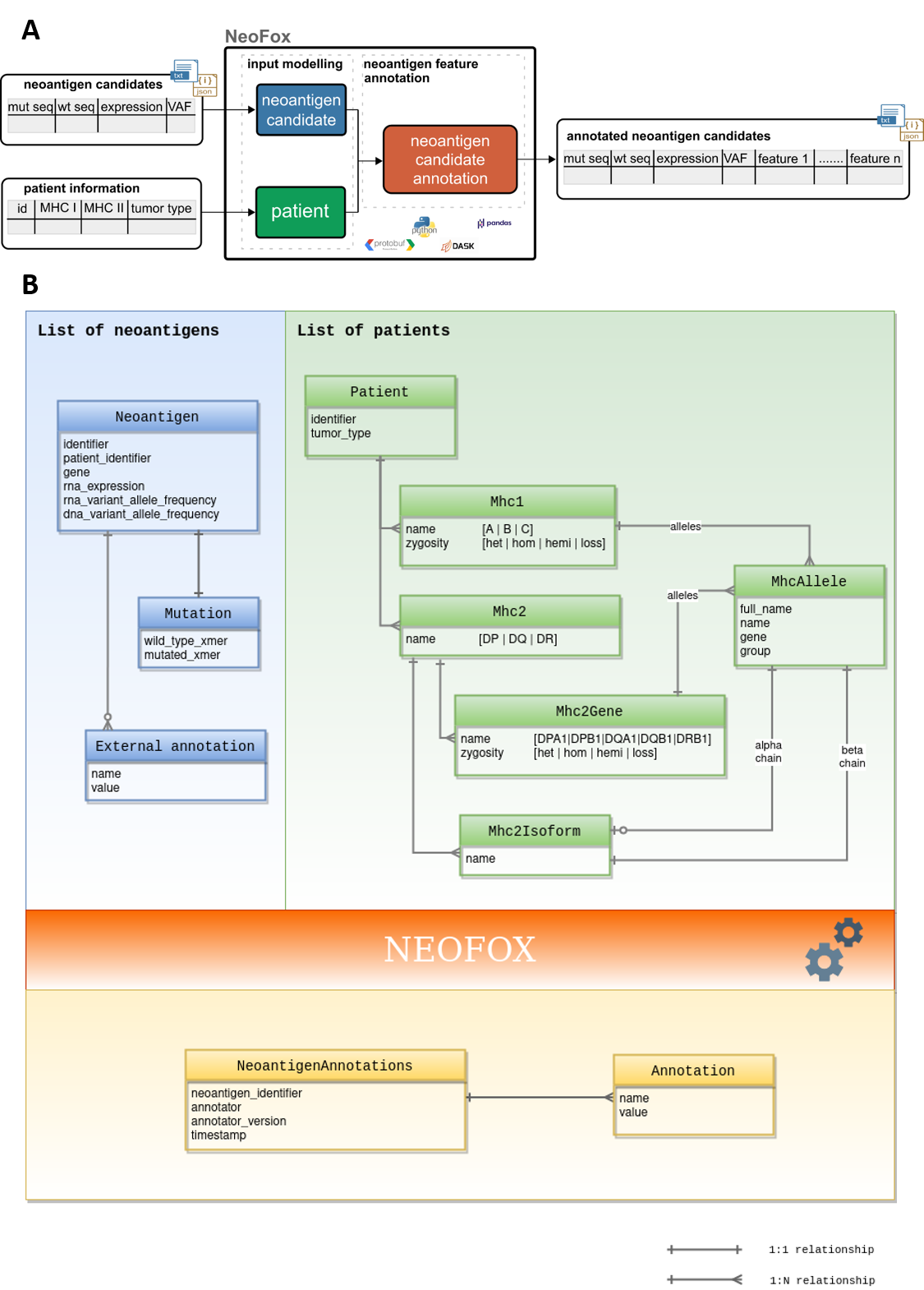
**

**Supplementary Figure S1**: **A)** General overview of NeoFox and its input and output. NeoFox requires two inputs by the users: neoantigen candidates and patient information. The input data is serialized into the internal models (see **B**) and neoantigen candidates are annotated with various features. NeoFox returns neoantigen candidates with the annotated feature values as output. Tabular and JSON formats are supported. **B**) Overview of data models representing the biological entities neoantigen candidates, patients and MHC I/II alleles in a machine-readable format. Protocol buffers was used to define structured and relational models for these entities. Each model (or in Protocol buffers language “message type”) is conceived as a minimal representation describing the given biological entity. A neoantigen candidate is defined by mutation, expression and optionally further external annotations. Likewise, patients and MHC genotypes are modelled in a structured manner. The model *Mhc1* reflects a MHC I gene and its allelic specification. The model *Mhc2* reflects a MHC II protein, defined by the list of involved MHC II genes, as modelled by *Mhc2Gene*, and list of allele specifications that form the MHC II heterodimer considering combinations of beta and alpha chains (*Mhc2Isoform*). Finally, neoantigen candidate annotations link each provided neoantigen candidate with a list of feature annotations (*NeoantigenAnnotations*). Annotations are stored as key-value pairs to maximize flexibility. In order to foster reproducibility of results, the annotations also contain metadata to track the different software versions and timestamps.


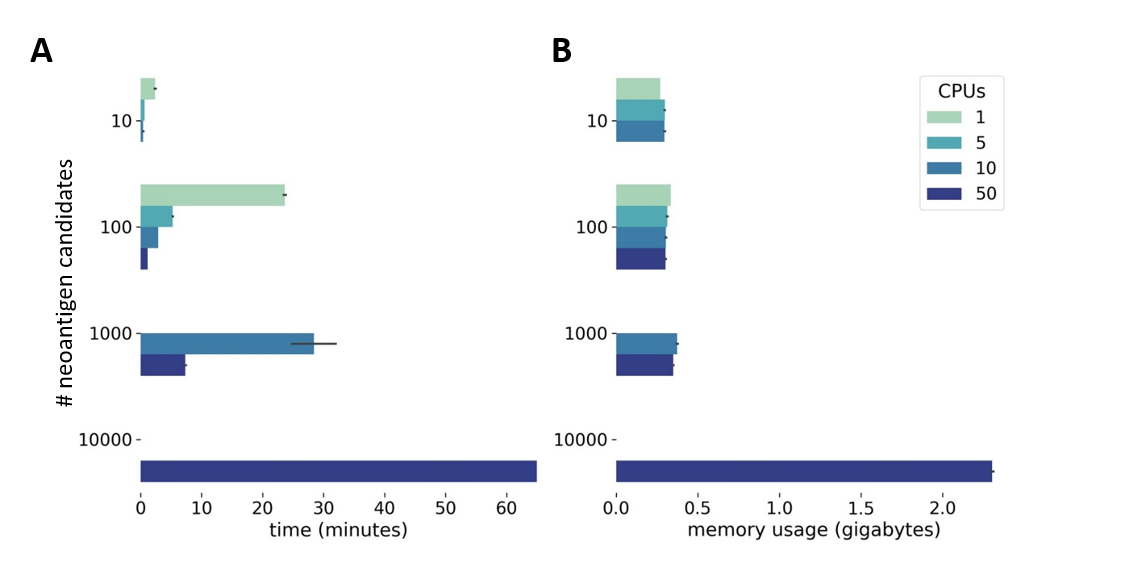


**Supplementary Figure S2**: Performance evaluation of NeoFox. **A)** Run time and **B**) memory usage was estimated for 10, 100, 1,000 and 10,000 neoantigen candidates using 1, 5, 10 or 50 CPUs. Barplots show the mean (+/- standard deviation) of 5 replicates for 10 to 1,000 datasets and 3 replicates for the dataset covering 10,000 datapoints. CPU: central processing unit.

References

Balachandran, V.P. *et al.* (2017) Identification of unique neoantigen qualities in long-term survivors of pancreatic cancer. *Nature*, **551**, 512 EP -.

Bassani-Sternberg, M. *et al.* (2017) Deciphering HLA-I motifs across HLA peptidomes improves neo-antigen predictions and identifies allostery regulating HLA specificity. *PLOS Computational Biology*, **13**, e1005725.

Besser, H. *et al.* (2019) Level of neo-epitope predecessor and mutation type determine T cell activation of MHC binding peptides. *Journal for ImmunoTherapy of Cancer*, **7**, 135.

Bjerregaard, A.-M. *et al.* (2017a) An Analysis of Natural T Cell Responses to Predicted Tumor Neoepitopes. *Frontiers in Immunology*, **8**, 1566.

Bjerregaard, A.-M. *et al.* (2017b) MuPeXI. Prediction of neo-epitopes from tumor sequencing data. *Cancer Immunology, Immunotherapy*, **66**, 1123–1130.

Calis, J.J.A. *et al.* (2013) Properties of MHC Class I Presented Peptides That Enhance Immunogenicity. *PLOS Computational Biology*, **9**, e1003266.

Duan, F. *et al.* (2014) Genomic and bioinformatic profiling of mutational neoepitopes reveals new rules to predict anticancer immunogenicity. *The Journal of Experimental Medicine*, **211**, 2231.

Gfeller, D. *et al.* (2018) The Length Distribution and Multiple Specificity of Naturally Presented HLA-I Ligands. *The Journal of Immunology*, ji1800914.

Ghorani, E. *et al.* (2017) Differential binding affinity of mutated peptides for MHC class I is a predictor of survival in advanced lung cancer and melanoma. *annonc*, **29**, 271–279.

Jensen, K.K. *et al.* (2018) Improved methods for predicting peptide binding affinity to MHC class II molecules. *Immunology*, **154**, 394–406.

Jurtz, V. *et al.* (2017) NetMHCpan-4.0. Improved Peptide–MHC Class I Interaction Predictions Integrating Eluted Ligand and Peptide Binding Affinity Data. *The Journal of Immunology*, **199**, 3360.

Kodysh, J. and Rubinsteyn, A. (2020) OpenVax: An Open-Source Computational Pipeline for Cancer Neoantigen Prediction. In Boegel, S. (ed), Bioinformatics for Cancer Immunotherapy: Methods and Protocols. New York, NY, Springer US, 147–160.

Łuksza, M. *et al.* (2017) A neoantigen fitness model predicts tumour response to checkpoint blockade immunotherapy. *Nature*, **551**, 517 EP -.

Marty, R. *et al.* (2017) MHC-I Genotype Restricts the Oncogenic Mutational Landscape. *Cell*, **171**, 1272-1283.e15.

Marty Pyke, R. *et al.* (2018) Evolutionary Pressure against MHC Class II Binding Cancer Mutations. *Cell*, **175**, 416-428.e13.

Racle, J. *et al.* (2019) Robust prediction of HLA class II epitopes by deep motif deconvolution of immunopeptidomes. *Nature Biotechnology*, **37**, 1283–1286.

Rech, A.J. *et al.* (2018) Tumor Immunity and Survival as a Function of Alternative Neopeptides in Human Cancer. *Cancer Immunology Research*, **6**, 276.

Richman, L.P. *et al.* (2019) Neoantigen Dissimilarity to the Self-Proteome Predicts Immunogenicity and Response to Immune Checkpoint Blockade. *Cell Systems*, **9**, 375-382.e4.

Rubinsteyn, A. *et al.* (2018) Computational Pipeline for the PGV-001 Neoantigen Vaccine Trial. *Frontiers in Immunology*, **8**, 1807.

Shen, W.-J., Wong, H.-S., Xiao, Q.-W., Guo, X. and Smale, S. (2012) Towards a Mathematical Foundation of Immunology and Amino Acid Chains. https://arxiv.org/abs/1205.6031.

Smith, C.C. *et al.* (2019) Machine-Learning Prediction of Tumor Antigen Immunogenicity in the Selection of Therapeutic Epitopes. *Cancer Immunology Research,* 2019: 10.1158/2326-6066.CIR-19-0155.
